# Crustacean methyl farnesoate–binding protein is an insect juvenile hormone–binding protein homolog that inhibits molting

**DOI:** 10.1016/j.jbc.2025.110297

**Published:** 2025-05-27

**Authors:** Hao Yang, Ting Chen, Xin Zhang, Mingyu Zhou, Lvping Zhang, Aifen Yan, Weihao Chen, Guiling Tan, Jingxuan Liang, Chunhua Ren, Xiaoli Chen, Zhi Li, Yao Ruan, Jiaxi Li, Hongmei Li, Peng Luo, Yanhong Wang, Xiao Jiang, Jiayue Yin, Bo Ma, Chunhua Zhu, Xugan Wu, Jiquan Zhang, Chaoqun Hu

**Affiliations:** 1 Laboratory of Zoological Systematics and Application of Hebei Province, College of Life Sciences, Hebei University, Baoding, China; 2 Key Laboratory of Breeding Biotechnology and Sustainable Aquaculture, Laboratory of Tropical Marine Bio-resources and Ecology, Guangdong Provincial Key Laboratory of Applied Marine Biology, South China Sea Institute of Oceanology, Chinese Academy of Sciences, Guangzhou, China; 3 Research Centre on Aquaculture Nutrition and Environmental Ecology of the Ministry of Agriculture and Rural affair, Shanghai Ocean University, Shanghai, China; 4 School of Medicine, Foshan University, Foshan, China; 5 State Key Laboratory of Cardiovascular Disease, Fuwai Hospital, Chinese Academy of Medical Sciences, Shenzhen, China; 6 Guangdong Research Center on Reproductive Control and Breeding Technology of Indigenous Valuable Fish Species, Fisheries College, Guangdong Ocean University, Zhanjiang, China

**Keywords:** methyl farnesoate–binding protein, methyl farnesoate, juvenile hormone, crustacean, penaeid shrimp, development

## Abstract

Methyl farnesoate (MF) serves as an essential regulator of key developmental processes in crustaceans, similar to juvenile hormones (JHs) in insects. However, it is susceptible to degradation in circulation. Despite the detection of proteins binding to MF in crustacean hemolymph for 3 decades, the precise genes encoding these proteins remain unclear. The present study identifies genes in crustaceans containing the juvenile hormone–binding protein (JHBP) domain. Among the 11 JHBPs found in the Pacific white shrimp (*Litopenaeus vannamei*), XP_027209752.1, which is primarily expressed in the hepatopancreas, emerges as the predominant form. This protein exhibits selective binding to MF rather than to JHs, leading to its designation as a methyl farnesoate–binding protein (MFBP) in crustaceans. Alterations in the amino acid sequence are predicted to induce structural changes that enhance the affinity of MFBP for MF. Endogenous MFBP inhibits molting, consistent with the function of MF. Furthermore, positive regulation of MFBP by MF has been observed in hepatopancreatic primary cells, with similar trends of hepatopancreatic MFBP mRNA and hemolymph MF levels during molting and ovarian development. This study identifies a novel MFBP in crustaceans that, despite being a homolog of insect JHBP, specifically binds MF to regulate molting in penaeid shrimp. These findings may advance our understanding of crustacean endocrine regulation and provide a molecular basis for improving their aquaculture techniques.

Juvenile hormones (JHs) represent a family of sesquiterpenoids that regulate development, reproduction, and metamorphosis in insects (1), with JH III being the most ubiquitous chemical form (2). Biosynthesis of JH begins with acetyl-CoA, which is converted to farnesyl diphosphate through the bilaterian-conserved mevalonate pathway, and then to farnesoic acid (FA) by enzymes farnesol (FN) phosphatase, FN dehydrogenase, and farnesal dehydrogenase, which are specific to arthropods (3). FA is further converted to methyl farnesoate (MF) in other groups of arthropods by juvenile hormone acid O-methyltransferase, or to JH in insects by juvenile hormone acid O-methyltransferase and JH epoxidase (CYP15A1/C1) (4, 5).

JHs are remarkably hydrophobic molecules, susceptible to facile degradation by esterases (6). As a safeguard, they associate with juvenile hormone–binding proteins (JHBPs) for protection against metabolic breakdown (7). Reported JHBPs include hemolymph JHBP (hJHBP) (8, 9), nuclear JHBP (10) and cytosolic JHBP (11), which are distinguished by their localization. The typical hJHBP contains the JHBP domain (IPR010562) (12) and is involved in protection of most JH molecules secreted from the corpora allata and delivered to target tissues (13). In contrast, nuclear JHBP and cytosolic JHBP might participate in intracellular JH signal transduction (10, 11).

The exclusive synthesis of JHs within the insect linage of arthropods is associated with the acquisition of JH epoxidase (3). In contrast, JHs have not been detected in crustaceans. Instead, MF, the exogenous nonepoxidized form or precursor of JH III, is considered an endogenous analog that regulates a range of developmental and physiological processes, similar to the role of JHs in insects (14, 15). In crustaceans, MF is synthesized by the mandibular organ, which is homologous to the corpora allata in insects (16). The roles of MF include stimulation of protein synthesis, sex determination, and regulation of the molt cycle and reproduction in both males and females (14, 17, 18).

Proteins with binding affinity for MF have been detected in the hemolymph and other tissues of various crustacean species, including the lobster *Homarus americanus* (19), crayfish *Procambarus clarkia* (20), crab *Cancer magister* (21) and spider crab *Libinia emarginata* (22). These proteins vary in size and are presumed to function as binding, carrier, or receptor proteins. However, the precise gene encoding the crustacean MF-binding protein remains unidentified. On the other hand, with the completion of genomic sequencing in crustacean species (23, 24, 25, 26), numerous crustacean genes containing JHBP domains have been found (27). However, JHs, which they bind to in insects, do not exist in crustaceans, and their functions remain unclear.

The Pacific white shrimp, *Litopenaeus vannamei*, is the most commercially significant species in global Penaeid shrimp aquaculture (23). Understanding the endocrine systems of economically important crustaceans like *L*. *vannamei* can contribute to the improvement of their aquaculture techniques (28, 29, 30). Here, we reported 11 JHBP domain–containing proteins identified by screening the genome of *L*. *vannamei*, with XP_027209752.1 identified as the predominant form among them. Subsequently, we validated the MF binding capability of XP_027209752.1, designating it as a novel methyl farnesoate–binding protein (MFBP) in crustaceans, and illustrated its protein structure and variation to transform the binding capacity between JH and MF. In addition, we elucidated the roles of MFBP in regulation of molting in *L*. *vannamei*. This study offers fresh insights into the MF-mediated development of Penaeid shrimp and contributes to the evolutionary differences of JH–MF system between insects and crustaceans and establishes a theoretical basis for regulating shrimp molting through MF.

## Results

### Cross-genomic and phylogenetic analysis of crustacean JHBP domain–containing genes

**Figure 1:**
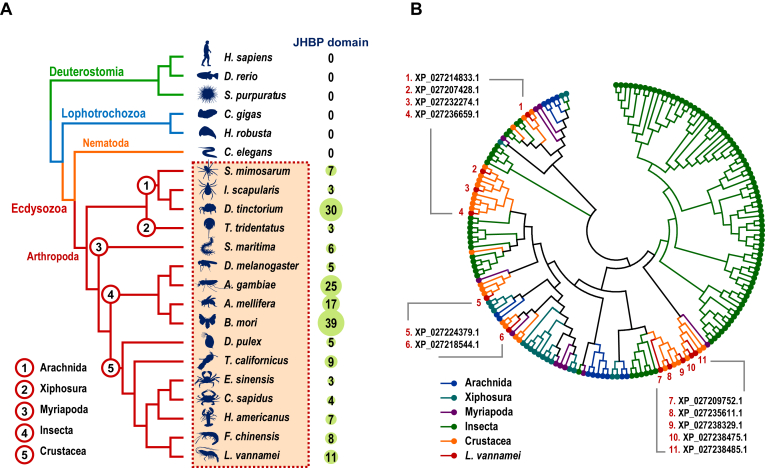
**JHBP domain-containing genes across species.***A*, the number of JHBP domain–containing genes in 22 species across different taxa (Deuterostomia, Lophotrochozoa, and Ecdysozoa) identified by InterProScan with genomic data (Table S3); *B*, phylogenetic analysis of JHBP domain–containing genes from the 22 species using neighbor-joining method with 1000 bootstrap replicates.

Based on cross-genomic analysis, the JHBP domains were only present in the species from Arthropoda within Ecdysozoa but were not found in any species from Deuterostomia, Lophotrochozoa, or Nematoda (Fig. 1*A* and Table S1). The numbers of JHBP domain–containing genes showed considerable variation across arthropod species, ranging from 3 to 39, whereas crustacean species exhibited a narrower range of 5 to 11 (Fig. 1*A*).

Phylogenetic analysis indicated that the JHBP domain–containing genes in arthropods were divided into two major branches. One branch was specific to Insecta, whereas the other branch was shared by Arachnida, Xiphosura, Myriapoda, Insecta, and Crustacea (Fig. 1*B* and S1).

### JHBP domain–containing gene XP_027209752.1 in *L*. *vannamei*

**Figure 2:**
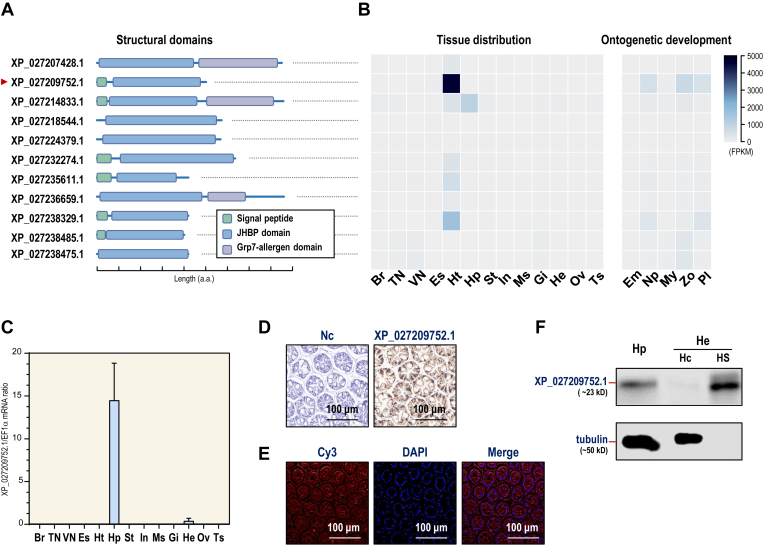
**JHBP domain-containing gene expression and localization in *L. vannamei.****A*, the structural domain organization of JHBP domain–containing genes in *Litopenaeus vannamei* annotated by InterProScan. *B*, expression profiles of JHBP domain–containing genes in various tissues and ontogenetic development stages by transcriptomic analysis. The selected tissues (n = 3, biological replicates) include the brain (Br), thoracic nerve (TN), ventral nerve cord (VN), eyestalk (Es), heart (Ht), hepatopancreas (Hp), stomach (St), intestines (In), muscle (Ms), gill (Gi), hemocytes (He), ovary (Ov), and testis (Ts). The ontogenetic development stages (n = 3, biological replicates) include embryo (Em), nauplius (Np), zoea (Zo), mysis (My), and postlarvae (Pl). *C*, the relative mRNA ratios of XP_027209752.1 among different tissues by quantitative PCR. *D*, *in situ* hybridization of XP_027209752.1 mRNA in the hepatopancreas to indicate positive signal and the nucleus (NC). *E*, immunofluorescent cellular localization of XP_027209752.1 protein in the hepatopancreas, with the protein and nucleus stained by antibody with Cy3 (*red*) and DAPI (*blue*), respectively. *F*, Western blot analysis of XP_027209752.1 protein in the hepatopancreas (Hp) and hemolymph (He). In He, the protein was detected in hemocytes (Hc) and hemolymph serum (HS). Tubulin was used as a loading control..

A total of 11 JHBP domain–containing genes were identified from the genome of *L*. *vannamei*, in which eight of them contain the JHBP domain solely, and three of them contain a Grp7-allergen domain followed by the JHBP domain (Fig. 2*A*). By analysis of the transcriptomic data, *XP_027209752*.*1* was shown to be the dominant form of *L*. *vannamei* JHBP domain–containing genes (Fig. 2*B*), and its transcript expression was restricted to the hepatopancreas of the adult shrimp (Fig. 2*C*). In addition, its expression increased during ontogenetic development (Fig. S2 and Table S2).

Upon confirmation by rapid amplification of complementary DNA ends, the ORF of *XP_027209752*.*1* is 648 bp, encoding a 215 amino acid protein precursor that includes a 16 amino acid signal peptide and a 199 amino acid mature protein with a 171 amino acid JHBP domain (Fig. S3). *In situ* hybridization (ISH) demonstrated that the *XP_027209752*.*1* transcript was expressed in all hepatopancreatic cells, and the protein signal was further characterized by immunofluorescence (IF; Fig. 2, *D* and *E*). In addition, Western blot analysis revealed the protein product of *XP_027209752*.*1* in the hemolymph serum but not in the hemocytes (Fig. 2*F*), indicating that it is a hepatopancreas-derived, hemolymph circulating protein.

### Ligand binding of XP_027209752.1 protein (MFBP)

**Figure 3:**
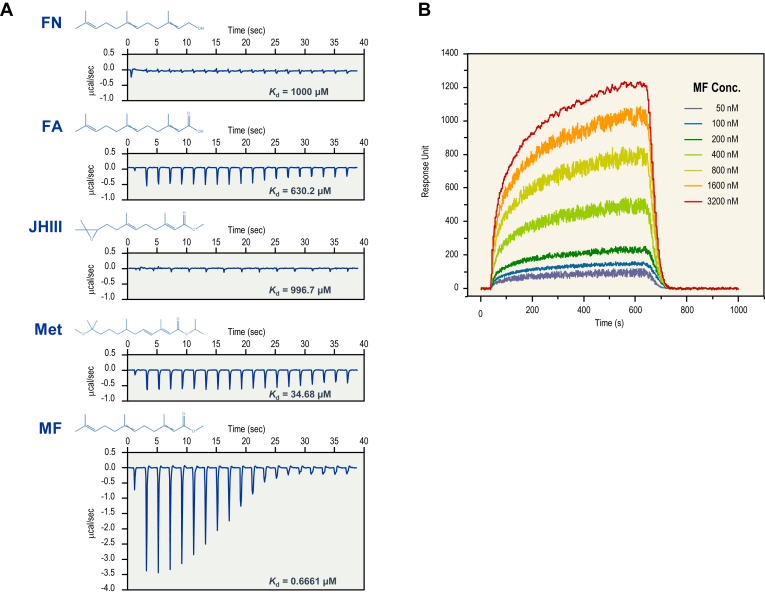
**Interaction analysis of r-*Lv*MFBP with ligands.***A*, isothermal titration calorimetry (ITC) analysis of ligand (200 μM) binding of r-*Lv*MFBP (20 μM). Raw injection profiles are shown for ligand screening, including FA (farnesoic acid), F-OH (farnesol), JH III (juvenile hormone III), Met (methoprene), and MF (methyl farnesoate). The corresponding *K*_*d*_s derived from nonlinear regression analysis are shown for all ligands. Data were processed with NanoAnalyze software with sequential fitting to independent models. *B*, surface plasmon resonance (SPR) analysis of the interactions between MF and r-*Lv*MFBP at concentrations of 50 to 3200 nM. r-*Lv*MFBP, recombinant form of *Litopenaeus vannamei* methyl farnesoate–binding protein.

To identify binding partners of JHBP domain–containing gene *XP_027209752*.*1*, the recombinant protein was generated in an *Escherichia coli* prokaryotic expression system (Fig. S4) and used to screen potential ligands (Fig. S5) through isothermal titration calorimetry and surface plasmon resonance (SPR). In this case, strong binding of XP_027209752.1 protein was demonstrated to MF, the exogenous nonepoxidized form of JH III (Fig. 3*A*). This binding was found to be highly specific, as only weak bindings were observed with FA and methoprene (Met), and no binding was detected with JH III and FN (Fig. 3*A*). By SPR, an increasing binding signal (response unit) was observed with the addition of MF to the immobilized XP_027209752.1 protein (Fig. 3*B*), confirming that MF is an exogenous ligand for XP_027209752.1. Thus, we designated XP_027209752.1 as a novel crustacean MFBP.

### Structure and binding mechanism of MFBP

**Figure 4:**
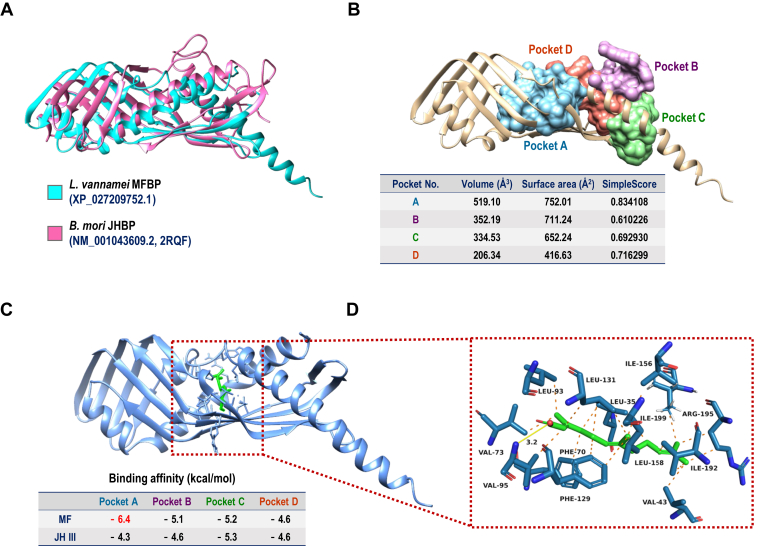
**Structural analysis of *Lv*MFBP and ligand binding pockets.***A*, comparison of the structures of *Lv*MFBP predicted by AlphaFold2 and *Bm*JHBP (NM_001043609.2, Protein Data Bank ID: 2RQF). *B*, four binding pockets of *Lv*MFBP predicted to interact with MF. *C*, binding of two hormones, MF and JH III, to pocket A. *D*, amino acids in pocket A of MFBP with the potential to bind MF. JH, juvenile hormone; *Lv*MFBP, *Litopenaeus vannamei* methyl farnesoate–binding protein; MF, methyl farnesoate; MFBP, methyl farnesoate–binding protein.

The 3D structures of *L*. *vannamei* MFBP (*Lv*MFBP) were predicted using AlphaFold2 (Fig. S6), and Model-1, the best model with 85% accuracy (Fig. S7), was selected for molecular docking. Using the crystal structure of *Bombyx mori* JHBP (Protein Data Bank ID: 2RQF) as a reference, *Lv*MFBP was successfully aligned and superimposed, with some significant deviations in the C terminus (Fig. 4*A*). Several potential binding pockets within *Lv*MFBP were detected, and the first four were ranked according to their sizes, surface areas, and simple scores (Fig. 4*B*). MF generally exhibited better docking than JH III in the four pockets with MFBP, especially in pocket A, which had the best binding affinity of −6.4 kcal/mol (Fig. 4*C*). In this case, MF molecule was docked inside a hydrophobic area surrounding by 13 residues within 4 Å and formed a 3.2 Å hydrogen bond with residue VAL-95 (Fig. 4*D*).

### Effects of MFBP silence on shrimp molting

**Figure 5:**
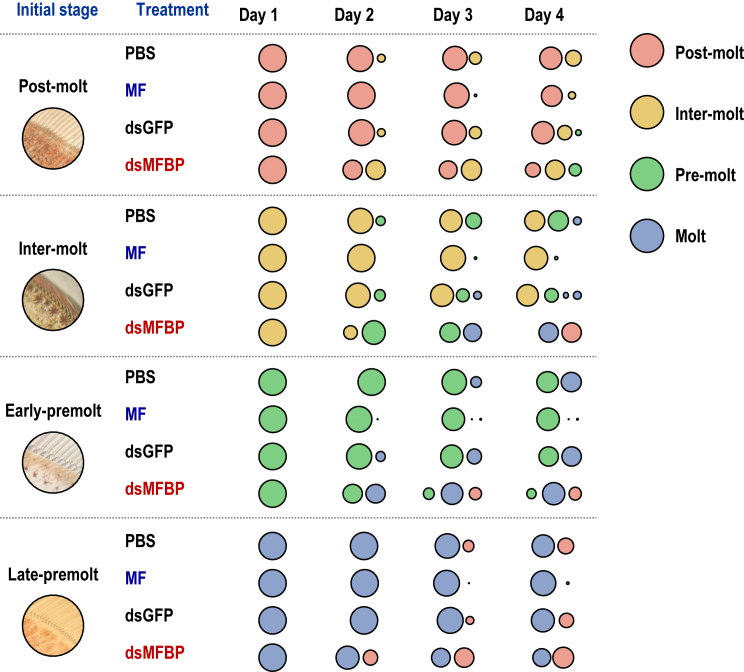
**Changes in molting pattern after RNAi of *Lv*MFBP and injection of MF**. Molting stages including postmolt (P1 and P2 stages), intermolt (C stage), early-premolt (D0, D1, and D2 stages), and late-premolt (D3 and D4 stages) were monitored for 24-h intervals following dsRNA injection (2 μg/g body weight). The circle sizes represent the number of individuals at this molting stage (n = 24, biological replicates). *Lv*MFBP, *Litopenaeus vannamei* methyl farnesoate–binding protein; MF, methyl farnesoate.

The effects of MFBP in shrimp molting were further investigated using an RNAi approach. The hepatopancreatic *Lv*MFBP mRNA was efficiently knocked down by injection of targeted dsRNA (Fig. S8). Molting of *L*. *vannamei* was accelerated in the groups silenced for *Lv*MFBP compared with those in the PBS and dsGFP injection groups (Fig. 5). On the contrary, MF injection delayed the molting of *L*. *vannamei*, indicating that natural *Lv*MFBP may inhibit molting by binding to MF.

### MF content and MFBP expression during molting and ovarian development

**Figure 6:**
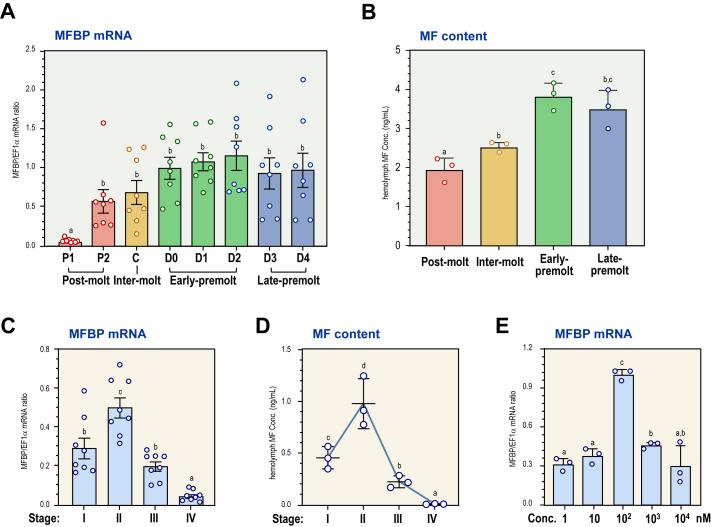
**MFBP mRNA and MF content regulation.***A*, expression profiles of *Lv*MFBP mRNA throughout the molting cycle, including postmolt (P1 and P2 stages), intermolt (C stage), early-premolt (D0, D1, and D2 stages), and late-premolt (D3 and D4 stages). *B*, MF contents measured by GC–MS during the molting cycle (n = 3, biological replicates). *C*, *Lv*MFBP mRNA during ovarian development, including stages I–IV (n = 4, biological replicates). *D*, MF contents during ovarian development (n = 3, biological replicates). *E*, regulation of *Lv*MFBP transcript by MF in the hepatopancreatic primary cells (n = 3, biological replicates). The data presented here are expressed as mean ± SE. Experimental groups denoted by the same letter represent a similar level of transcript expression (*p* < 0.05, ANOVA followed by Dunnett’s multiple comparisons test). *Lv*MFBP, *Litopenaeus vannamei* methyl farnesoate–binding protein; MF, methyl farnesoate.

During molting, the transcripts of *Lv*MFBP increased from P1 at postmolt stage to D0 at premolt stage, remained at high levels until D4 at late-premolt stage, and then sharply decreased once molting occurred (Fig. 6, *A* and *B*). The hemolymph serum MF contents and hepatopancreatic *Lv*MFBP transcripts were measured at different stages during shrimp ovarian development (Fig. S9). The expression of *Lv*MFBP transcripts and MF contents followed a highly similar trend, increasing from stage I, peaking at stage II, then continuously decreasing to reach the lowest levels at stage IV (Fig. 6, *C* and *D*).

### Regulation of MFBP transcript by MF

The regulation of *Lv*MFBP transcript by MF was demonstrated in hepatopancreatic primary cells (Fig. 6*E*). Upon a fixed incubation duration of 24 h, MF stimulated the expression of *Lv*MFBP across concentrations ranging from 1 to 100 nM in a dose-dependent manner. However, this stimulatory effect diminished when the concentration increased to 1000 nM, indicating that MF positively regulates the expression of *Lv*MFBP at physiological concentrations.

## Discussion

JH and MF are sesquiterpenoids that regulate crucial physiological and developmental processes in insects and crustaceans (4). They are unstable molecules that require protection by binding proteins during transportation in the hemolymph (31). Following the initial discovery of the insect JHBP gene in the tobacco hornworm *Manduca sexta* (8), proteins with the capacity to bind to MF were subsequently detected for the first time in crustaceans (19). These proteins range in size from 29 to 116 kDa (19, 20, 21, 22); however, the precise genes encoding these proteins remain undetermined to date.

This study is the first to report that the crustacean MFBP, which possesses a high binding capacity for MF but not JHs, is a homologous gene of the insect hJHBP featuring a typical JHBP domain (IPR010562). In insects, JHBP exhibits high binding affinities to the JHs, JH I, JH II, and JH III, but not to nonepoxidized analogs, such as MF (32). Structurally, *Lv*MFBP displays binding pockets (Fig. 4*B*) that are distinct from those of insect hJHBP (12). Molecular modeling and docking analyses predict that 13 residues are critical for *Lv*MFBP to form a binding pocket with a structural preference for MF over JHs (Fig. 4*C*), suggesting that amino acid substitution is an essential step in the transition of JHBP binding affinity from JH ligands. The evolution of the insect JH pathway from the pancrustacean ancestors has been previously attributed to the appearance of the MF epoxidase enzyme, CYP15A1 (33), and an amino acid mutation in the JH receptor, Met tolerant (34). Our current study offers new insights on the protection of hemolymph JH by the alterations of insect JHBP from the ancestral pancrustacean MFBP, thus completing the molecular description of the evolutionary history of the JH pathway, encompassing synthesis, transport, and reception.

In crustaceans, molting and reproduction are highly intricate processes delicately regulated by a series of hormones (35, 36). The roles of MF include the promotion of molting and gonadal development in both males and females (37). Our study demonstrated a correlation between hepatopancreatic *Lv*MFBP transcript levels and hemolymph MF concentration (Fig. 6, *A*, *C* and *D*), a relationship attributable to the positive regulation of MFBP by MF (Fig. 6*E*). Furthermore, the silencing of *Lv*MFBP mRNA was shown to accelerate the molting cycle (Fig. 6*A*), suggesting that natural MFBP may inhibit crustacean molting, and this effect may be mediated by binding to MF. In insects, JHBP regulates development by binding to JH (38), similar to the role of MFBP and MF in crustaceans, as demonstrated in our study. Although the ligands for these homologous binding proteins are different, they share functional similarities with their common ancients.

Several crustacean species in order Decapoda are economically important in global aquaculture (39), and understanding their endocrine systems can improve culture techniques. Although decapod crustaceans belong to arthropods, their divergence time from insects spans evolutionarily separated periods of up to 400 million years (33). Thus, the identification of functional genes in decapod crustaceans should be based on their own genomes (23, 24, 40, 41, 42), rather than by comparison to related species. In addition, gene insertions, deletions, and fusions have occurred in the crustacean genomes (43). Consequently, protein motifs/domains from InterPro member databases (44) were chosen for the identification of JHBP homologs in crustaceans, instead of a BLAST-based gene annotation (45). Furthermore, expansions of many gene families have been observed in the crustacean genomes (23). The predominant form of JHBP domain–containing genes (*XP_027209752*.*1*) was determined by comparing their transcript levels through transcriptomic sequencing, and it was confirmed to be MFBP by molecular biological methods.

In conclusion, we have first identified a novel MFBP gene in a crustacean species. This study demonstrated the affinity between MFBP and MF and illustrated the structural foundations and evolutionary mechanism of crustacean MFBPs in comparison with their insect counterparts, JHBPs. We also investigated the inhibitory effect of MFBP on molting in crustacean. The present study, therefore, provides new insights into the protection and transportation of MF in this crustacean-specific pathway and introduces a way for novel gene identification in crustaceans.

## Experimental procedures

### Animals and sample collection

Adults, embryos, and larvae of the Pacific white shrimp were collected from the Jinyang Shrimp Culture Center in Maoming, Guangdong, China and maintained in artificial seawater (30 parts per thousand [ppt] and pH 8.2) at 28°C under a 12-h dark–12-h light photoperiod. Tissue samples were collected from adult shrimp (average weight = 35.67 ± 6.24 g). Hemolymph was extracted from the pericardial sinus using hypodermic needles with trisodium citrate dihydrate as an anticoagulant, and the hemocytes and hemolymph serum were separated by centrifugation at 1000*g*. For ontogenetic development, samples were collected from embryos, nauplius, zoea, mysis, and postlarvae (46). For molting, adult shrimp (average weight = 6.04 ± 1.32 g) were observed, and the molting cycle was determined as previously described (47). For ovarian development, previtellogenic female shrimp (average weight = 54.02 ± 8.74 g) were artificially induced to undergo ovarian maturation by unilateral eyestalk ablation and nutritional enhancement (48). The ovarian developmental stages were categorized as previtellogenesis (stage I), primary vitellogenesis (stage II), secondary vitellogenesis (stage III), and maturation (stage IV), as previously described (49). All shrimp experiments were conducted in accordance with the guidelines of the Animal Research and Ethics Committees of the South China Sea Institute of Oceanology, Chinese Academy of Sciences.

### Cross-genomic identification and phylogenetic analysis of JHBP domain–containing genes

JHBP domain–containing genes were obtained from the genomes of 22 animal species, including *Homo sapiens*, *Danio rerio*, and *Strongylocentrotus purpuratus* as representatives of Deuterostomia; *Crassostrea gigas* and *Homarus robusta* as Lophotrochozoa; *Caenorhabditis elegans* as Nematoda of Ecdysozoa; and *Stegodyphus mimosarum*, *Ixodes scapularis*, and *Dinothrombium tinctorium* as Arachnida, *Tachypleus tridentatus* as Xiphosura, *Strigamia maritima* as Myriapoda, *Drosophila melanogaster*, *Anopheles gambiae*, *Apis mellifera*, and *Bombyx mori* as Insecta of Arthropoda in Ecdysozoa. Within Arthropoda, *Daphnia pulex*, *Tigriopus californicus*, *Eriocheir sinensis*, *Callinectes sapidus*, *Homarus americanus*, *Fenneropenaeus chinensis*, and *L*. *vannamei* were included as Crustacea (Table S3). These sequences were systematically scanned using InterProScan (v5.27-66.0) with default parameters to identify proteins containing the JHBP domain (InterPro accession: IPR010562). The analysis was performed in domain architecture mode, targeting exclusively to the JHBP domain. To confirm the absence of JHBP domains in nonarthropod taxa, all protein sequences from these species were subjected to the same workflow.

Phylogenetic analysis of JHBPs was conducted based on the amino acid sequences of the JHBP domains in the JHBP domain–containing genes identified across genomes using MEGA (v6.0). The neighbor-joining method (pairwise deletion) with 1000 bootstrap replicates was used for the analysis.

### RNA sequencing and gene expression analyses of JHBP domain–containing genes

Transcript levels of *L*. *vannamei* JHBP domain–containing genes in different tissues (SRX10331229—SRX10331241) and during ontogenetic developmental stages (SRX625463, SRX625466, SRX625467, SRX625474, and SRX625475) were analyzed through RNA sequencing. Paired-end clean reads were aligned to the *L*. *vannamei* reference genome (23) by using HISAT2 (v2.1.0). Transcripts were assembled, and read counts for each gene were calculated using StringTie (v1.3.5). Counts per million mapped reads were calculated, and cross-sample normalization was performed using DESeq2 and demonstrated as a heatmap by using TBtools.

### Molecular cloning and recombinant protein generation

Total RNA was extracted from the *L*. *vannamei* hepatopancreas using TRIzol reagent (Invitrogen), digested with DNase I (Invitrogen), and reverse transcribed into first-strand complementary DNA using the PrimeScript II Kit (Takara). The ORF of XP_027209752.1 was determined by PCR with gene specific primers, and the full-length sequence was obtained by rapid amplification of complementary DNA end. The primer sequences used in this study are listed in Table S4.

The recombinant protein of XP_027209752.1 (r-*Lv*MFPB) was expressed in *E*. *coli* (BL21-T1R), purified using a HisTALON Gravity Column (Clontech), and desalted using a PD-10 Desalting Column (GE Healthcare).

### ISH and IF

For ISH, a 530-bp antisense DNA probe for XP_027209752.1 (Table S6) was labeled with digoxigenin. The ISH signal was developed using the diaminobenzidine method by incubation with horseradish peroxidase–conjugated antidigoxigenin antibody, and the nuclei were restained with hematoxylin (50).

For IF, a polyclonal antibody against *Lv*MFBP (XP_027209752.1) was purified from the serum of a rabbit injected with recombinant *Lv*MFBP protein as the antigen. The antibody was labeled with Cy3 dye (532 nm; Invitrogen) for visualization of the endogenous protein, and 4′,6-diamidino-2-phenylindole reagent (305 nm; Invitrogen) was used for staining of the cell nuclei (50).

### Western blot

Total protein was extracted from the hepatopancreas, hemocytes, and hemolymph serum using a protein extraction kit (Sangon Biotech). Western blot analysis of *Lv*MFBP (XP_027209752.1) was performed as previously described (49). In this case, tubulin was used as an internal control, and the images were visualized with a C-DiGit Blot Scanner (LI-COR).

### Isothermal titration calorimetry

Isothermal titration calorimetry experiments were conducted using an IC200 Microcalorimeter (Malvern Panalytical) at 30 °C. The r-*Lv*MFBP protein and ligands (FN, FA, JH III, Met, and MF) were each dissolved in 20 mM Tris–HCl (pH 7.5) at concentrations of 20 μM and 200 μM, respectively. The ligands were delivered in 2 μl injections to the protein samples at 150-s intervals. Baseline corrections were accomplished by titrating the protein into sample buffer, and the injection enthalpies calculated were analyzed using the MicroCal Origin program. The titration data were analyzed using NanoAnalyze software (TA Instruments) with sequential fitting to independent models, and the dissociation constants (*K*_*d*_) values were calculated based on data fitting.

### Surface plasmon resonance

SPR experiments were conducted using a Biacore T200 (GE Healthcare). The r-*Lv*MFBP protein was dissolved in PBST at pH 7.4 (0.1% Tween-20) with concentrations ranging from 50 μM to 3200 μM. Glycine–HCl (pH 2.0) was used for regeneration. The association phase lasted 600 s, and the dissociation phase lasted 360 s. After subtracting the reference and buffer signals, the data were fitted to both a steady-state binding model and a 1:1 kinetic model to determine the *K*_*d*_ and k_off using the Biacore T200 Evaluation software (GE Healthcare).

### Molecular docking and dynamics simulation

Molecular docking analysis was conducted using the open-source program AutoDock Vina (51). Five models of *Lv*MFBP were predicted by AlphaFold (version 2.1.1) (52), and Model-1, which was identified as the best model, was chosen as the receptor. The structures of MF and JH III as ligands were generated using ChemBio Office (version 17.0) and subsequently optimized through an MM2 calculation to minimize their conformational energy. Both the ligands and receptor were converted to PDBQT format using AutoDock Tools (version 1.5.6). Four potential active sites were identified as grid boxes with the help of an online tool, DoGSiteScorer (53). The docking pose with the lowest binding energy was considered the most favorable binding conformation and was visually analyzed using either UCSF Chimera (version 1.15) or PyMOL (version 2.4).

### RNA interference

*Lv*MFBP dsRNA was synthesized using a T7 RiboMAX Express RNAi System (Promega). A total of 96 shrimp were randomly assigned to three groups, each with 24 individuals. In the experimental group, shrimp were injected with dsMFBP (2 μg/g body weight) in PBS, and controls included injections of dsEGFP in PBS or PBS alone. The experimental group was also compared with another group injected with MF (2 μg/g body weight). Molting stages of the shrimp were observed and recorded four times at 24-h intervals using a microscope as previously described (47).

### Culture and treatment of hepatopancreatic primary cells

Shrimp hepatopancreatic primary cells were prepared and cultured as previously described (54). In the experiment, MF at concentrations ranging from 1 to 10^4^ nM was added to the culture medium as treatments. After 24 h of incubation, the culture media were removed and the cells were harvested for the measurement of *Lv*MFBP mRNA.

### Quantitative real-time PCR

Transcript expression of *Lv*MFBP was detected in various tissues, as well as in different ontogenetic, ovarian developmental, and molting stages using quantitative real-time PCR. The reactions were performed using SYBR Premix Ex Taq II (TaKaRa) with specific primers under certain conditions (Table S4 and S5), and elongation factor 1-α (GU136229.1) was used as the internal control. Normalized data expressed as mean ± SE were analyzed by using one-way ANOVA followed by Dunnett’s multiple comparisons test, and differences were considered as significant at *p* < 0.05.

### Sesquiterpenoid hormone measurement

GC–MS was utilized to analyze the content of MF. Hemolymph samples from *L*. *vannamei* during distinct molting stages and ovarian developmental stages were collected and extracted, then adjusted to a final volume of 100 μl with hexane. Chromatographic separation and mass spectrometric detection were conducted using the Trace1300-TSQ9000 GC–MS system (Thermo Scientific). Sample injection into the chromatograph was done using a microsyringe, and MF contents were analyzed in the selected ion monitoring mode, with specific ions *m/z* 114 used as a quantitative marker and *m/z* 41 and 69 as qualitative markers. Analytes were ionized in the mass spectrometer postseparation on the chromatographic column, followed by analysis through the mass analyzer and detection by the detector, with automatic data acquisition and processing.

## Data availability

The data that support the findings of this study are available in the Experimental procedures section and supporting information of this article.

## Supporting information

This article contains supporting information (23, 47, 48)

## Conflict of interest

The authors declare that they have no conflicts of interest with the contents of this article.
