# Supplementary material for: Crustacean methyl farnesoate–binding protein is an insect juvenile hormone–binding protein homolog that inhibits molting
Source: J Biol Chem. 2025 May 27;301(7):110297. doi: 10.1016/j.jbc.2025.110297 (PMC12256323; doi:10.1016/j.jbc.2025.110297)
Supplement: Supporting Information [file mmc1.docx]

**Supplementary Information for**

**Crustacean Methyl Farnesoate Binding Protein (MFBP) is an Insect Juvenile Hormone Binding Protein Homolog (JHBP) that inhibits molting**

Hao Yang, Ting Chen ^*^, Xin Zhang, Mingyu Zhou, Lvping Zhang, Aifen Yan, Weihao Chen, Guiling Tan, Jingxuan Liang, Chunhua Ren, Xiaoli Chen, Zhi Li, Yao Ruan, Jiaxi Li, Hongmei Li, Peng Luo, Yanhong Wang, Xiao Jiang, Jiayue Yin, Bo Ma, Chunhua Zhu, Xugan Wu ^*^, Jiquan Zhang ^*^, Chaoqun Hu ^*^

This file includes:

Supplementary Figures S1 to S9.

**
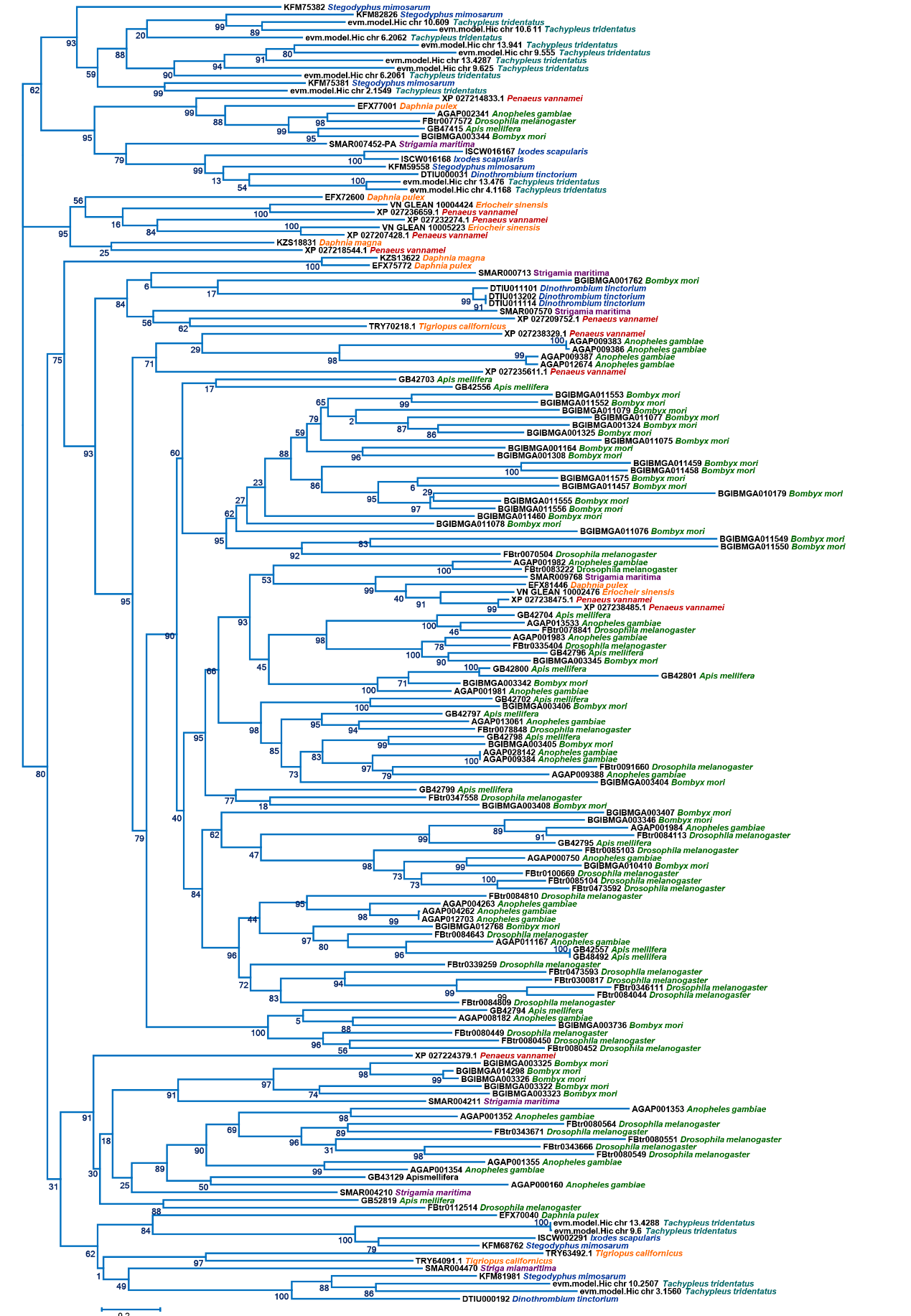
**

**Figure S1.** Phylogenetic analysis of JHBP domain-containing genes from the 22 species.


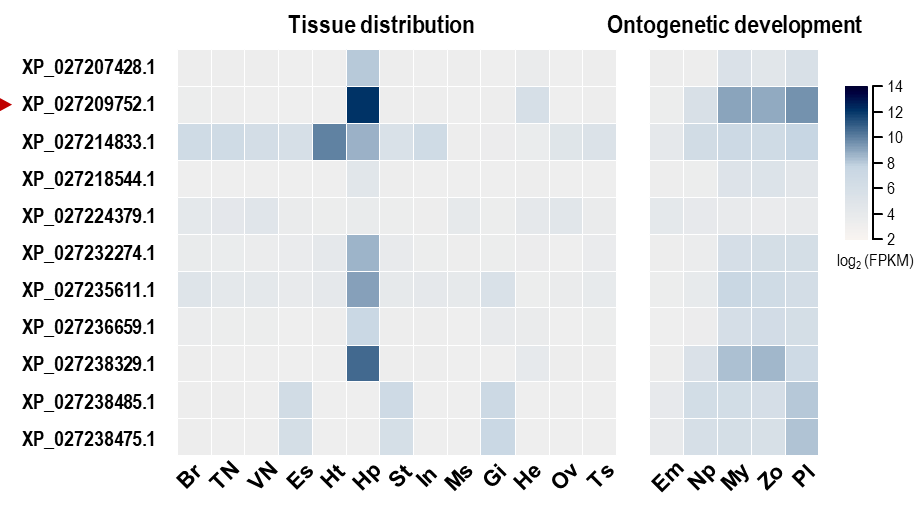


**Figure S2.** Expression profiles of JHBP domain-containing genes in various tissues and ontogenetic development stages by transcriptomic analysis. The gene expression levels in the heatmap are displayed with a log scale.

**
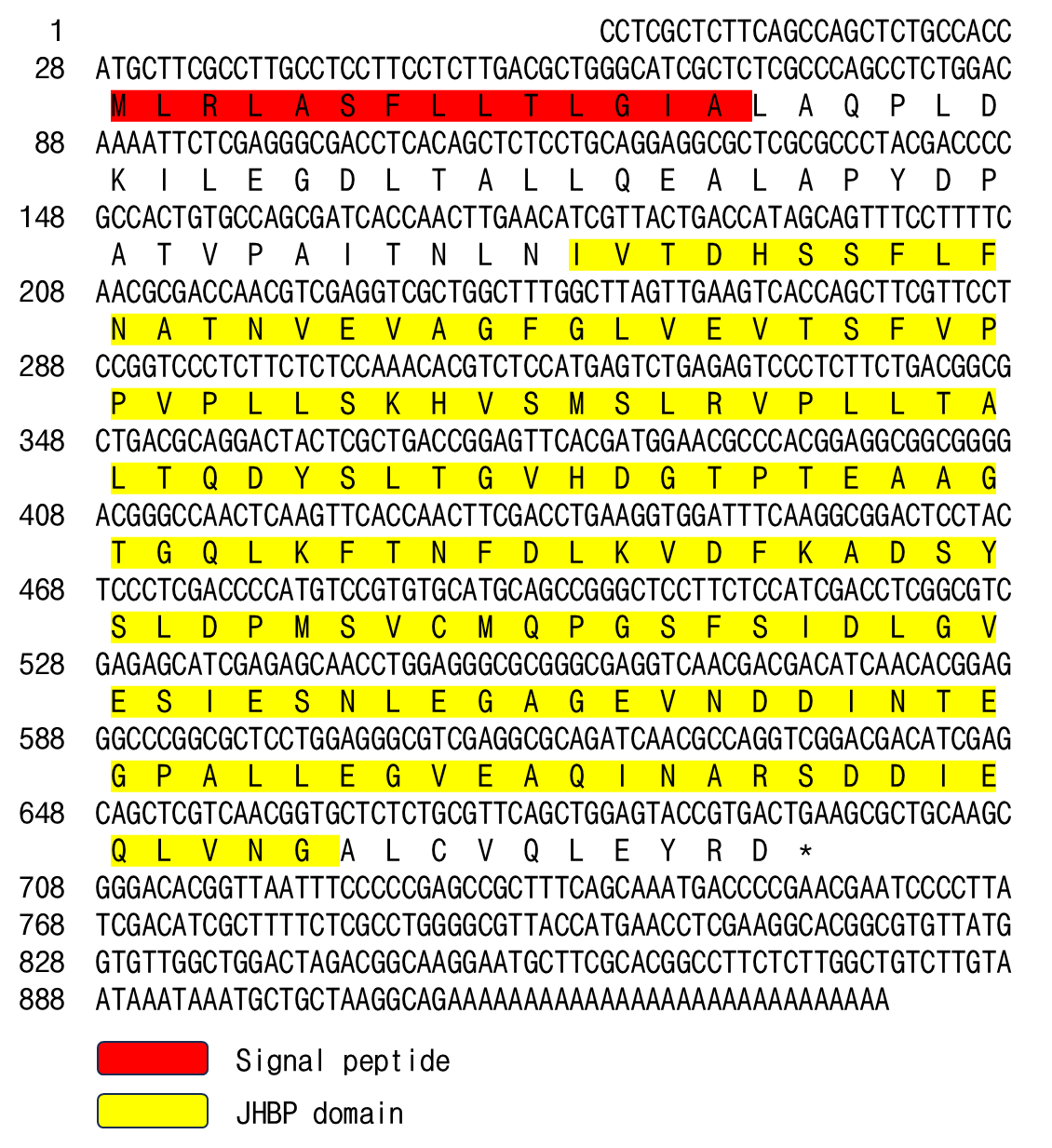
**

**Figure S3.** The cDNA corresponding amino acid sequences of *Lv*MFBP (XP_027209752.1).


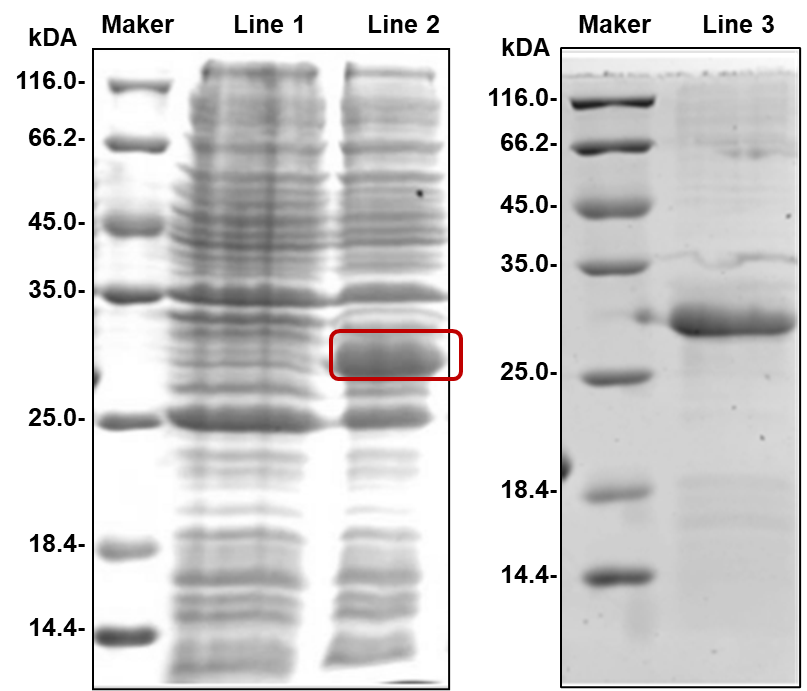


**Figure S4.** SDS-PAGE of the r-*Lv*MFBP protein. Line 1: Bacterial lysate without IPTG induction; Line 2: Bacterial lysate with IPTG induction; Line 3: Purified protein.

| **Farnesol (FN)** | 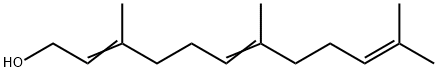 |
| --- | --- |
| **Farnesoic acid (FA)** | 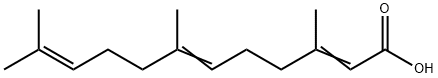 |
| **Juvenile hormone III (JH III)** | 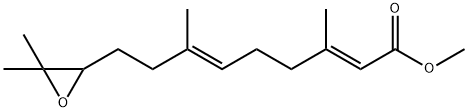 |
| **Methoprene (Met)** | 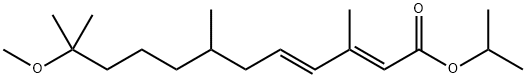 |
| **Methyl Farnesoate (MF)** | 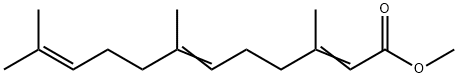 |

​**​**

**Figure S5.** Structural of five juvenile hormone (JH)-related compounds.​


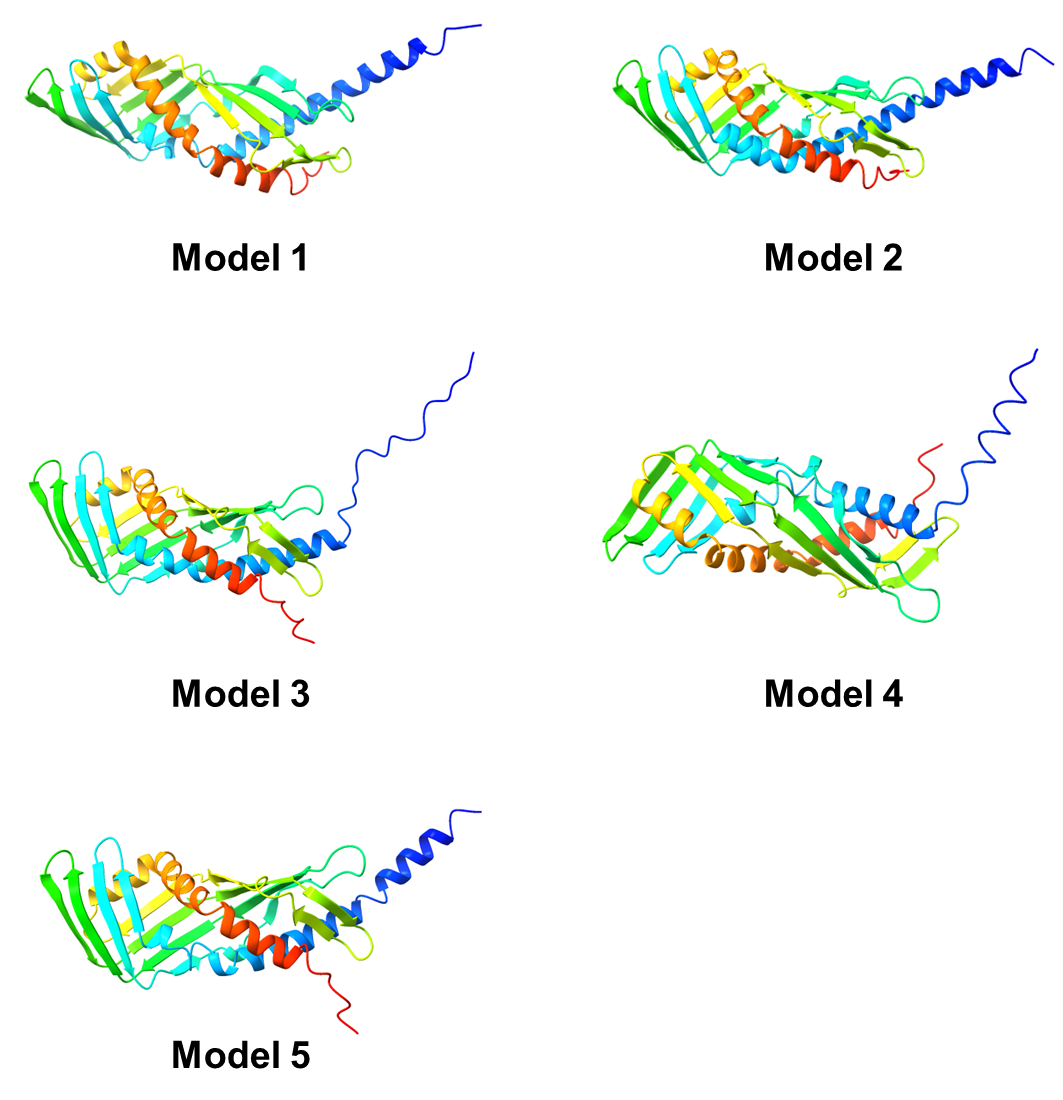


**Figure S6.** Protein structures of *Lv*MFBP (XP_027209752.1) as predicted with AlphaFold 2.0.

**
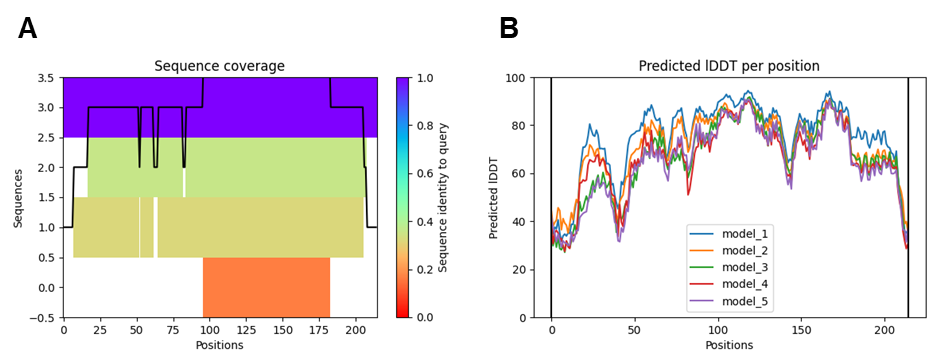
**

**Figure S7. A:** Alphafold sequence coverage score. Black curve shows the number of sequences found at each residue position, so the horizontal axis shows coverage of protein from C terminus to N terminus. Vertical axis indicates Sequence identity to query as red color shows low sequence identity, green and blue show higher sequence identity. **B:** The prediction confidence scores of 5 models built by AlphaFold (v2.1.1) were evaluated based on IDDT. Confidence thresholds were defined as very high (lDDT > 90), confident (90 > lDDT > 70), low (70 > lDDT > 50), and very low confidence (lDDT < 50).


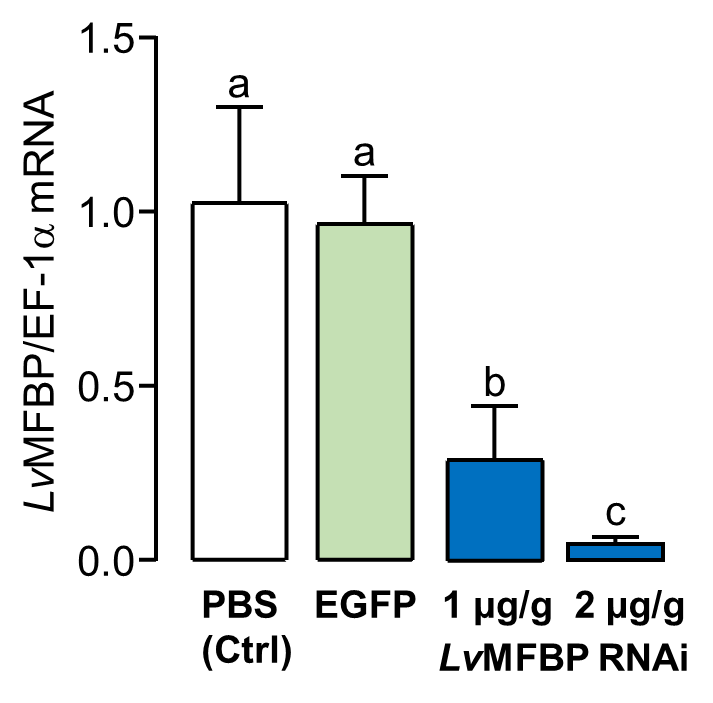


**Figure S8.** Hepatopancreatic *Lv*MFBP mRNA expression after injection of PBS (blank control), dsRNA of EGFP (negative control), or dsRNA of *Lv*MFBP for 12 h. Identically assigned letters represent a comparable level of transcriptional expression (*P*> 0.05), while differently assigned letters represent significant difference in levels of transcriptional expression between two groups (*P*< 0.05, one-way ANOVA followed by Dunnectt’s multiple comparisons test).

**
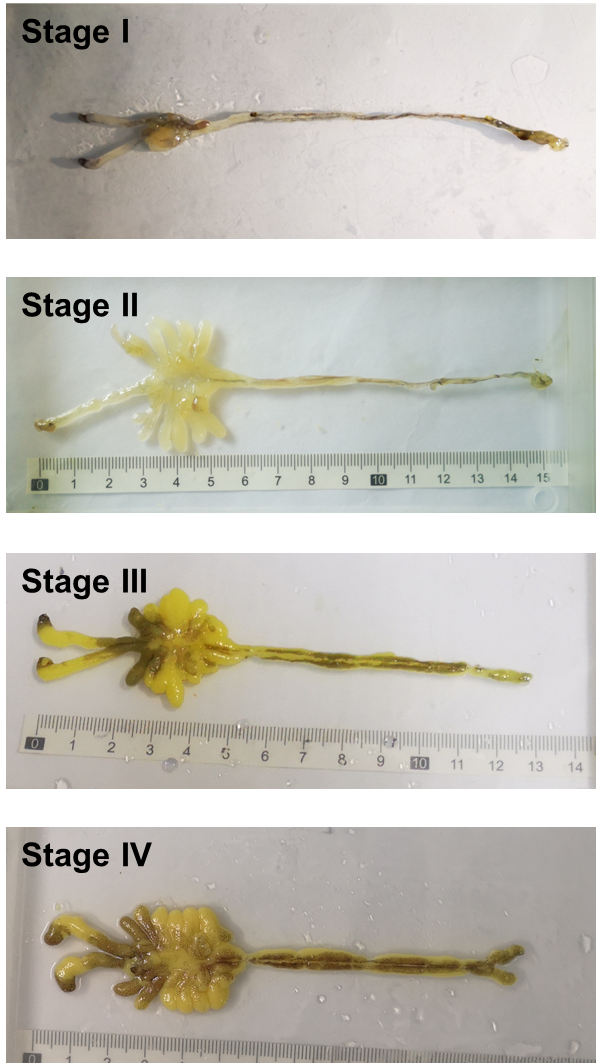
**

**Figure S9.** Ovarian morphologies of *L. vannamei* at different ovarian developmental stages.
